# A novel non-invasive exhaled breath biopsy for the diagnosis and screening of breast cancer

**DOI:** 10.1186/s13045-023-01459-9

**Published:** 2023-06-16

**Authors:** Jiaqi Liu, Haibin Chen, Yalun Li, Yanman Fang, Yang Guo, Shuangquan Li, Juan Xu, Ziqi Jia, Jiali Zou, Gang Liu, Hengyi Xu, Tao Wang, Dingyuan Wang, Yiwen Jiang, Yang Wang, Xuejie Tang, Guangdong Qiao, Yeqing Zhou, Lan Bai, Ran Zhou, Can Lu, Hongwei Wen, Jiayi Li, Yansong Huang, Shuo Zhang, Yong Feng, Hongyan Chen, Shouping Xu, Bailin Zhang, Zhihua Liu, Xiang Wang

**Affiliations:** 1 Department of Breast Surgical Oncology, National Cancer Center/National Clinical Research Center for Cancer/Cancer Hospital, Chinese Academy of Medical Sciences and Peking Union Medical College, 17 Panjiayuannanli, Chaoyang, Beijing, 100021 People’s Republic of China; 2 State Key Laboratory of Molecular Oncology, National Cancer Center /National Clinical Research Center for Cancer/Cancer Hospital, Chinese Academy of Medical Sciences and Peking Union Medical College, 17 Panjiayuannanli, Chaoyang, Beijing, 100021 People’s Republic of China; 3 Breax Laboratory, PCAB Research Center of Breath and Metabolism, Beijing, 100071 People’s Republic of China; 4 Department of Breast Surgery, The Affiliated Yantai Yuhuangding Hospital of Qingdao University, Yantai, 264000 People’s Republic of China; 5 Department of Breast Surgery, Guiyang Maternal and Child Healthcare Hospital, Guiyang, 550001 People’s Republic of China; 6 Department of Breast Surgery, Yanqing Maternal and Child Healthcare Hospital of Beijing, Beijing, 101399 People’s Republic of China; 7 Department of Radiology, The First Affiliated Hospital of Wenzhou Medical University, Wenzhou, 325000 People’s Republic of China; 8 Department of Breast Surgery, Daxing Maternal and Child Healthcare Hospital of Beijing, Beijing, 100162 People’s Republic of China; 9 Peking Union Medical College and Chinese Academy of Medical Sciences, Beijing, 100005 People’s Republic of China; 10 Department of Neurosurgery, Xuanwu Hospital, China International Neuroscience Institute, National Center for Neurological Disorders, Capital Medical University, Beijing, 100053 People’s Republic of China; 11 Department of Breast Surgery, The Fourth Hospital of Hebei Medical University, Shijiazhuang, 050019 Hebei People’s Republic of China; 12 Department of Breast Surgery, Harbin Medical University Cancer Hospital, Harbin, 150081 People’s Republic of China

**Keywords:** Breast cancer, Breath test, Volatile organic compound, Early diagnosis, Cancer screening

## Abstract

**Background:**

Early detection is critical for improving the survival of breast cancer (BC) patients. Exhaled breath testing as a non-invasive technique might help to improve BC detection. However, the breath test accuracy for BC diagnosis is unclear.

**Methods:**

This multi-center cohort study consecutively recruited 5047 women from four areas of China who underwent BC screening. Breath samples were collected through standardized breath collection procedures. Volatile organic compound (VOC) markers were identified from a high-throughput breathomics analysis by the high-pressure photon ionization–time-of-flight mass spectrometry (HPPI-TOFMS). Diagnostic models were constructed using the random forest algorithm in the discovery cohort and tested in three external validation cohorts.

**Results:**

A total of 465 (9.21%) participants were identified with BC. Ten optimal VOC markers were identified to distinguish the breath samples of BC patients from those of non-cancer women. A diagnostic model (BreathBC) consisting of 10 optimal VOC markers showed an area under the curve (AUC) of 0.87 in external validation cohorts. BreathBC-Plus, which combined 10 VOC markers with risk factors, achieved better performance (AUC = 0.94 in the external validation cohorts), superior to that of mammography and ultrasound. Overall, the BreathBC-Plus detection rates were 96.97% for ductal carcinoma in situ, 85.06%, 90.00%, 88.24%, and 100% for stages I, II, III, and IV BC, respectively, with a specificity of 87.70% in the external validation cohorts.

**Conclusions:**

This is the largest study on breath tests to date. Considering the easy-to-perform procedure and high accuracy, these findings exemplify the potential applicability of breath tests in BC screening.

**Supplementary Information:**

The online version contains supplementary material available at 10.1186/s13045-023-01459-9.

**To the editor**

Breast cancer (BC) is one of the most common cancers and a leading cause of death worldwide [1]. Early BC detection improves survival [2]. However, imaging-based BC screening methods are prone to being expensive and overdiagnosed. [3] By detecting volatile organic compounds (VOCs) during exhalation [4], breath biopsy is a promising non-invasive strategy for early cancer detection [5]. However, the accuracy of the breath test for BC diagnosis has not been verified by multi-center clinical trials with sufficient sample sizes [4].

Herein, we enrolled 5047 women who underwent BC screening from six hospitals in four areas of China (Fig. 1 and Additional file 1: Figure S1). The discovery set included 216 BC patients and 2959 non-cancer women from three hospitals in Beijing, and the external validation set included 249 BC patients and 1545 non-cancer women from another three hospitals in Yantai, Wenzhou, and Guiyang, respectively (Additional file 1: Tables S1, S2). Most BC patients were diagnosed at early stages (Additional file 1: Table S3).

**Fig. 1:**
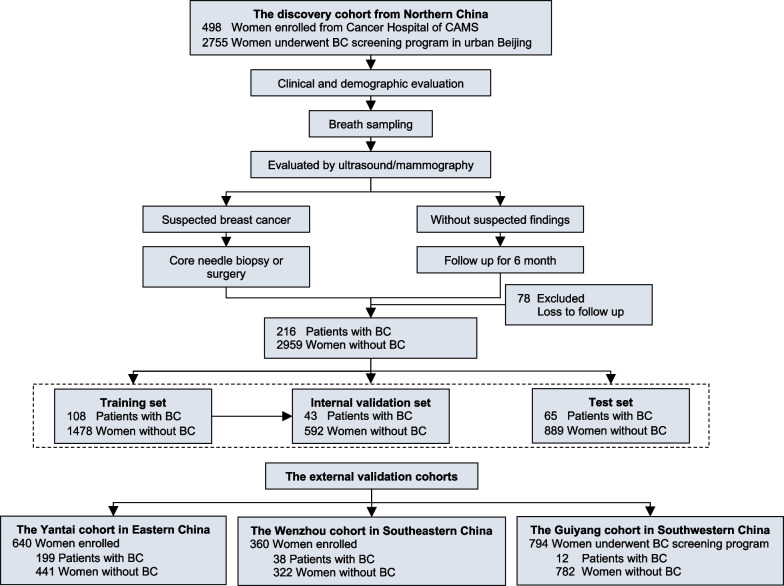
**Patient Enrollment and Study Design.** This multi-center cohort study consecutively recruited women who underwent breast cancer screening at six hospitals in China. The participants were divided into the discovery cohort to identify candidate VOCs and to construct diagnostic models, and the external validation cohorts to independently test the diagnostic value of the models. In the model construction, the discovery dataset was randomly split into training, internal validation, and test datasets with a ratio of 5:2:3. The external validation cohorts enrolled women who underwent opportunistic breast cancer screening at Yantai and Wenzhou and women underwent the population-based breast cancer screening at Guiyang. For each participant, the information of risk factors for breast cancer and breath sample was breath sample collected before the standard mammography and ultrasonography. The final diagnosis was based on the pathology result and a 6-month follow-up. 78 patients lost to follow-up were excluded. Abbreviation: BC, breast cancer; CAMS, Chinese Academy of Medical Sciences

Breath samples of 1.2L for each participant were collected according to established procedures and analyzed by high-pressure photon ionization time-of-flight mass spectrometry (HPPI-TOFMS) (Additional file 1: Supplementary methods) [6]. HPPI-TOFMS has a higher throughput than earlier technologies and does not require the pretreatment of exhaled breath [7]. Each VOC ion’s peak area was then computed. Spectrum peak patterns and VOC correlation modules of the BC patients and controls differed (Additional file 1: Figures S2 and S3). Ten optimal VOC features were selected to differentiate the BC patients and non-cancer controls in the discovery cohort (Fig. 2A). Eight VOCs showed significantly higher peak areas in BC patients than controls, and two VOCs were substantially lower (Fig. 2B and Additional file 1: Table S4). Significant fold changes and diagnostic performances were identified in these 10 VOC ions (Additional file 1: Figure S4). The m/z values of 28.0 and 40.0, which may contain ethylene and propyne or fragment ions, showed the highest AUCs (Fig. 2C and Additional file 1: Table S4).

**Fig. 2:**
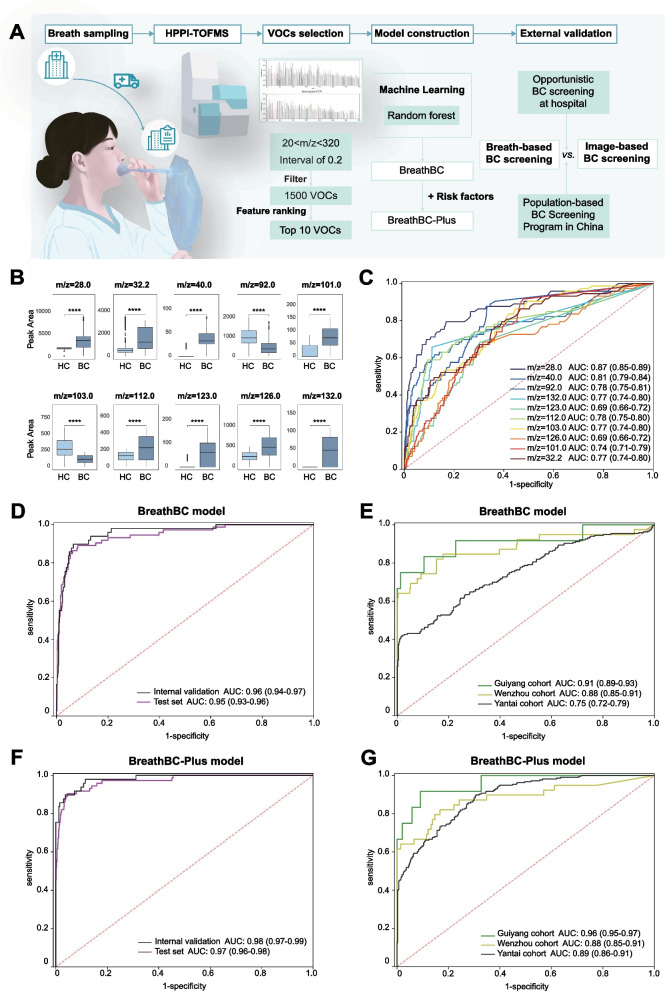
**The Workflow of Data Analysis, the Distribution of the Top Ten Volatile Organic Compound (VOC) Ions with High Contribution Coefficients in the Models Construction, and the Performance of the Breast Cancer Detection Models for the BreathBC Model and BreathBC-Plus Model.** **A** The workflow of data analysis and models construction. Breath samples were collected through standardized breath collection procedures using self-designed collectors and airbags and then analyzed by the high-pressure photon ionization–time-of-flight mass spectrometry (HPPI-TOFMS). Data for 1500 VOC ions were detected from the m/z range of [20, 320) with an interval of 0.2. Based on the random forest algorithm, the optimal 10 VOC ions were confirmed based on the feature importance or coefficient in the model training. Two breast cancer detection models (BreathBC and BreathBC-Plus) were constructed using the breath VOC markers with or without risk factors. Both models were verified with the three external validation cohorts. **B** Ten optimal VOC ions demonstrated significant differences between patients with breast cancer and non-cancer women among all the participants in this study, including eight elevated VOCs and two decreased VOCs. **C** The receiver operating characteristic (ROC) curves and the associated areas under curves (AUCs) of the diagnostic performance of the ten optimal VOC ions. **D**–**E** For the BreathBC model using 10 breath VOC markers, the diagnostic AUC was 0.96 (95% CI, 0.94–0.97) in the internal validation cohort, 0.95 (95% CI, 0.93–0.90) in the test cohort (**D**), and 0.87 in the external validation cohorts (**E**). **F**, **G** For the BreathBC-Plus model using both breath VOC markers and risk factors, the combined model performed better than the BreathBC model in the internal validation cohort and the test cohort (AUC = 0.97–0.98) (**F**) and the external validation cohorts (AUC = 0.94) (**G**). Abbreviation: AUC, areas under curve. Abbreviation: HPPI-TOFMS, high-pressure photon ionization–time-of-flight mass spectrometry; VOC, volatile organic compound; BC, breast cancer; HC, healthy control; AUC, areas under curve

The random forest algorithm [8] was employed as the classifier. The discovery dataset was randomly split 5:2:3 into training, internal validation, and test datasets for model construction. We constructed two BC detection models, BreathBC and BreathBC-Plus, using only the 10 VOC markers and both VOC markers and risk factors, respectively (Fig. 2A).

BreathBC scores were higher in BC patients than controls (0.66 ± 0.31 vs. 0.11 ± 0.15, *p* = 1.29 × 10^−153^), regardless of tumor size, lymph node status, and molecular subtypes (all *p* < 0.01, Additional file 1: Figure S5), and collinear with tumor size (r = 0.41, *p* = 0.05; Additional file 1: Figure S6). The diagnostic AUC of the BreathBC model was 0.96 (95%CI, 0.94–0.97) in the internal validation cohort and 0.95 (95%CI, 0.93–0.90) in the test cohort (Fig. 2D, E, Additional file 1: Table S5). The performances are higher than all the results of previous studies using the gas chromatography-mass spectrometry (GC–MS) (AUC = 0.67–0.93) [9–11] but lower than the electronic nose (AUC = 0.99; Additional file 1: Table S6) [12]. However, no external validation was conducted for the previous methods, and their sample sizes were relatively small. In external validation cohorts, the BreathBC model achieved an AUC of 0.87, a sensitivity of 92.37% (230/249), and a specificity of 60.45% (934/1545; Additional file 1: Table S7).

Furthermore, the BreathBC-Plus diagnostic model was developed in the discovery cohort, combining BreathBC scores with traditional risk factors (Additional file 1: Supplementary methods). The combined model outperformed the BreathBC model in the internal validation cohort (AUC = 0.98), the test cohort (AUC = 0.97), and external validation cohorts (AUC = 0.94) (Fig. 2F, G, Additional file 1: Table S5). In external validation cohorts, BreathBC-Plus produced sensitivity and specificity of 89.16% (222/249) and 87.70% (1355/1545; Additional file 1: Table S7). Collectively, the total detection rates were 96.97% (32/33) in ductal carcinoma in situ (DCIS), 85.06% (74/87), 90.00% (99/110), 88.24% (15/17), and 100% (2/2) for stages I, II, III, and IV BC in external validation cohorts, respectively (Additional file 1: Table S8). Intriguingly, breathBC-Plus outperformed mammography and ultrasound in diagnosis (Additional file 1: Figure S7, Table S9).

There are some limitations of this study. First, although the HPPI-TOFMS provided a high-throughput methodology for VOC analysis, it is still being determined which chemical compound is associated with each MS peak. Second, as most previous studies on VOCs were only focusing on one cancer type, we also aimed to identify the BC-specific VOC markers in this study.

To our knowledge, this is the largest breathomics analysis study to date. Collectively, breath-based methods may provide supplemental or alternative screening strategies to detect early-stage BC and DCIS at comparable performance to imaging-based technologies.

## Supplementary Information

**Additional file 1**.Supplementary Methods, Figures, and Tables. 

## Data Availability

The supplementary data supporting this study's findings are openly available in the supplemental materials. Deidentified participant data and analytic code are available upon reasonable request to Dr. Jiaqi Liu (j.liu@cicams.ac.cn).

## Abbreviations

AUC: Area under the curve
BC: Breast cancer
CI: Confidence interval
DCIS: Ductal carcinoma in situ
GC–MS: Gas chromatography–mass spectrometry
HC: Healthy control
HPPI-TOFMS: High-pressure photon ionization-time-of-flight mass spectrometry
ROC: Receiver operating characteristic
VOC: Volatile organic compound
