# Supplementary material for: A novel non-invasive exhaled breath biopsy for the diagnosis and screening of breast cancer
Source: J Hematol Oncol. 2023 Jun 16;16:63. doi: 10.1186/s13045-023-01459-9 (PMC10276488; doi:10.1186/s13045-023-01459-9)
Supplement: Supplementary file 1 — Additional file 1.Supplementary Methods, Figures, and Tables. [file 13045_2023_1459_MOESM1_ESM.pdf]

Supplementary material

Liu *et al*. A Novel Non-invasive Exhaled Breath Biopsy for the Diagnosis and Screening of Breast Cancer.

Supplementary methods

Study Design

This multicenter cohort study consecutively recruited women who underwent breast cancer screening from six hospitals in China, from December 19, 2020, to January 21, 2022. The participants were further divided into the discovery cohort to identify candidate VOCs and construct diagnostic models and the external validation cohorts to test the diagnostic value of these models. The discovery cohort enrolled women who underwent opportunistic breast cancer screening at the Cancer Hospital of Chinese Academy of Medical Sciences and Peking Union Medical College and women from the population-based breast cancer Screening Program in Urban Beijing[1] at Yanqing District and Daxing District, all in Beijing. The external validation cohorts enrolled women underwent opportunistic breast cancer screening at the Affiliated Yantai Yuhuangding Hospital of Qingdao University (the Yantai cohort in Eastern China) and the First Affiliated Hospital of Wenzhou Medical University(the Wenzhou cohort in Southeastern China), and women from the population-based Breast Cancer Screening Program in Urban China[2] at Guiyang (the Guiyang cohort in Southwestern China). The study was reviewed and approved by the ethics committee of each participating hospital. Written informed consent was obtained from each participant. This study followed the Standards for Reporting of Diagnostic Accuracy (STARD) reporting guidelines.[3]

Participants and Clinical Evaluation

In a screening setting, women underwent opportunistic breast cancer screening at age ≥18 years, and women involved in the population-based breast cancer Screening Program with age ≥30 years were consecutively recruited in this study. The exclusion criteria include (1) malignant tumor diagnosed within 5 years; (2) anticancer treatment within 5 years; (3) active respiratory infections; (4) inhalation anesthesia within 3 months; (5) treatment of inhaled medication within 3 months. For each participant, we collected the clinical diagnosis result, breath sample, as well as risk factors. The information of risk factors for breast cancer was collected for each participant, including age,

body mass index (BMI), family cancer history, personal cancer history, and menopause status.

Standard mammography and ultrasonography were conducted at each center. The images were interpreted with double reading and classified according to the classification standard of the breast imaging-reporting and data system (BI-RADS) by two experienced radiologists or sonographers at each center.[4,5] Women with breast lesions suspected of malignancy as BI-RADS category 4-5 were biopsied by surgery or core-needle biopsy afterward. The diagnosis of each patient was based on the pathology results. All women without suspicious breast lesions or with negative biopsy results were followed up every 3 months for 6 months until a final diagnosis was made. The molecular subtype was divided according to the pathologic criteria for hormone receptor (HR, including estrogen receptor /ER and progesterone receptor/PR) and ERBB2/HER2.[6] The ER, PR, HER2, and Ki-67 were measured by immunohistochemistry. The staging was determined according to the 8th edition of the classification for breast cancer by the American Joint Commission of Cancer (AJCC).[7]

Breath Sample Collection and Analysis

In this study, we set standard sampling demands and protocols to minimize the influence of daily diet, smoking, alcohol, environment, etc. Firstly, the participants were required to prepare for sampling in advance: no smoking, alcohol, or diet within an hour before sampling. Secondly, participants were required to rinse their mouths with purified water instantly before sampling to minimize the influence of diet, smoking, alcohol, etc. Thirdly, all samples are required to be collected at the same site, which could minimize the effects of environmental facts. With a deep nasal inhalation, participants completely exhaled the air into the sampling bag via a disposable gas nipple. The breath sampling bag with a volume of 1.2L is made of polyether-ether-ketone (PEEK).

HPPI-TOFMS, which consisted of a vacuum ultraviolet (VUV) lamp-based HPPI ion source and an orthogonal acceleration time-of-flight (TOF) mass analyzer, was used to detect and analyze the breath samples. The HPPI ion source was designed with two ionization models: soft HPPI ionization and collision-induced dissociation ionization. The soft-HPPI model is adopted in this study. It will predominantly produce radical cations (M^+), which are formed as $M + h\gamma \rightarrow M^+ + e$. A commercial VUV-Kr lamp with a photon energy of 10.6 eV and a photon flux of 1011 photons/s was adopted for the gas-phase sample ionization, which can ionize most VOCs in the breath. Breath samples were directly introduced through a 250 μm i.d. 0.60 m long stainless-steel

capillary. All the mass spectra were accumulated for 60 seconds. Then, the mass spectrum peaks with m/z <350 were detected and accumulated for 60 seconds via a time-to-digital converter in HPPI-TOFMS, its design and structure details were introduced in Wang's study[8] and Meng's study[9]. The noise-reducing and baseline correction were implemented for the original mass spectrogram via anti-symmetric wavelet transformation, which was achieved by the Python package pywavelets[10]. To transfer the discrete signal of mass spectra data to standard VOC features, the area of the most substantial peak in the range of [x-0.1, x+0.1) was calculated and regarded as the feature of VOC with m/z of x. Considering no signal detected for m/z<20 and m/z>320, 1500 VOCs ions data were detected from the m/z range of [20, 320) with an interval of 0.2, which were used for machine learning model construction.

Considering the qualitative ability of HPPI-TOF-MS is limited, we deduced the possible chemicals of the top ten breast cancer-related VOC ions based on their m/z, peak area, and the physicochemical property of potential biomarkers. Firstly, we search for possible breath metabolic components based on the m/z value from the human breath-omics database[11]. Then, we confirmed the most portable ions based on detectable concentrations, physicochemical properties, and published breast cancer VOC biomarkers.

Model Development

A statistical-based feature selection method was executed to select valuable VOC ions and avoid model over-fitting, and VOC ions with no significant differences (p >0.05) were excluded. As the machine learning model was constructed, the most critical VOC ions were confirmed based on the feature importance or coefficient in the machine learning model training. Peak area difference analysis was also performed on the relative density of the VOC ions between the breast cancer and control groups. The random forest (RF) algorithm was employed as the classifier for breast cancer detection.[12] The datasets of all the enrolled participants were divided into one discovery cohort and three external validation cohorts according to the areas of enrollment. The discovery dataset was randomly split as train, internal validation, and test data sets with a ratio of 5:2:3, which were used for model construction. The internal validation data set was for model validation and confirming the cut-off value to achieve the optimal sensitivity with a specificity no less than 60%. The remaining test data set was for testing with label blinding. In this study, we constructed two breast cancer detection models, BreathBC and BreathBC-Plus. BreathBC was

constructed using breath VOC data. BreathBC-Plus was constructed using breath VOC data and clinical risk factors, including age, body mass index (BMI), family cancer history, personal cancer history, and menopause status.

Statistical Analysis

Descriptive statistics were reported as frequency (percentage) for categorical variables or mean with standard deviation for continuous variables. We compared the demographics of different patient groups using the student's T test for continuous variables and the chi-square test for categorical variables. The sensitivity, specificity, positive and negative predictive value, accuracy, area under the receiver operating characteristic (ROC) curve (AUC), and relative 95% confidence interval (CI) were calculated to evaluate the performance of the VOC-based breast cancer detection models. The AUCs of the models were compared with the DeLong test.[13,14] The sample sizes were decided to verify that the AUC is non-inferior to the performance of traditional diagnostic imaging in the independent validation cohorts. Two-sided p-values less than 0.05 were considered statistically significant for all analyses. All statistical analyses were performed using SAS (version 9.4; SAS Institute Inc., USA), Origin software (version 2018; OriginLab Corporation, USA), PASS (version 2021; UCSS, USA), and R statistical software (version 3.5.1; The R Foundation, USA).

Sample Size Estimation

In this study, the minimum sample size was 12 for the breast cancer patients and 82 for the controls in each independent validation cohort to effectively verify that the performance (AUCs) of the combined model (the BreathBC-Plus) is non-inferior to the performance of traditional diagnostic imaging (AUC=0.80 for mammography and ultrasound[15]) with the power (1-Beta) of 90% and significance level (Alpha) of 0.05 using a two-sided z-test[16,17]. Thus, we finally enrolled 199 breast cancer patients and 441 women without breast cancer in the Yantai cohort, 38 breast cancer patients and 322 women without breast cancer in the Wenzhou cohort, and 12 breast cancer patients and 782 women without breast cancer in the Guiyang cohort. The sample size estimation was conducted by the PASS 2021 (UCSS, USA).

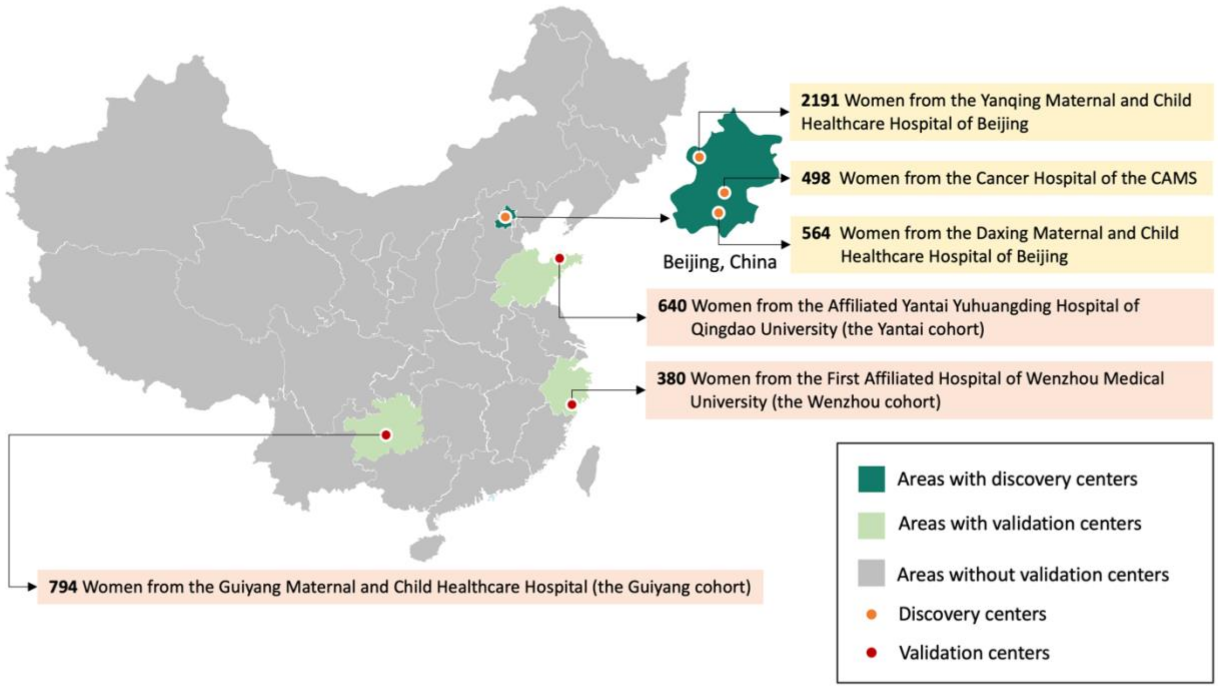

Figure S1. The Multi-Center Validation in Different Regions in China.

This multi-center cohort study consecutively recruited women who underwent breast cancer screening at six hospitals in China from December 19, 2020, to January 21, 2022. The participants were divided into the discovery cohort (dark green area) to identify candidate VOCs and construct diagnostic models, and the external validation cohorts (light green areas) to test the diagnostic value of the models. The discovery cohorts (orange dots) enrolled 498 women who underwent opportunistic breast cancer screening at the Cancer Hospital of the Chinese Academy of Medical Sciences and Peking Union Medical College and 2755 women from the population-based Breast Cancer Screening Program in Urban Beijing in the Yanqing District and Daxing District of Beijing (all in Beijing, Northern China). The external validation cohorts (red dots) enrolled 640 women who underwent opportunistic breast cancer screening at the Affiliated Yantai Yuhuangding Hospital of Qingdao University (the Yantai cohort in Eastern China) and 380 women from the First Affiliated Hospital of Wenzhou Medical University (the Wenzhou cohort in Southeastern China) and 794 women from the population-based Breast Cancer Screening Program in Urban China at Guiyang (the Guiyang cohort in Southwestern China).

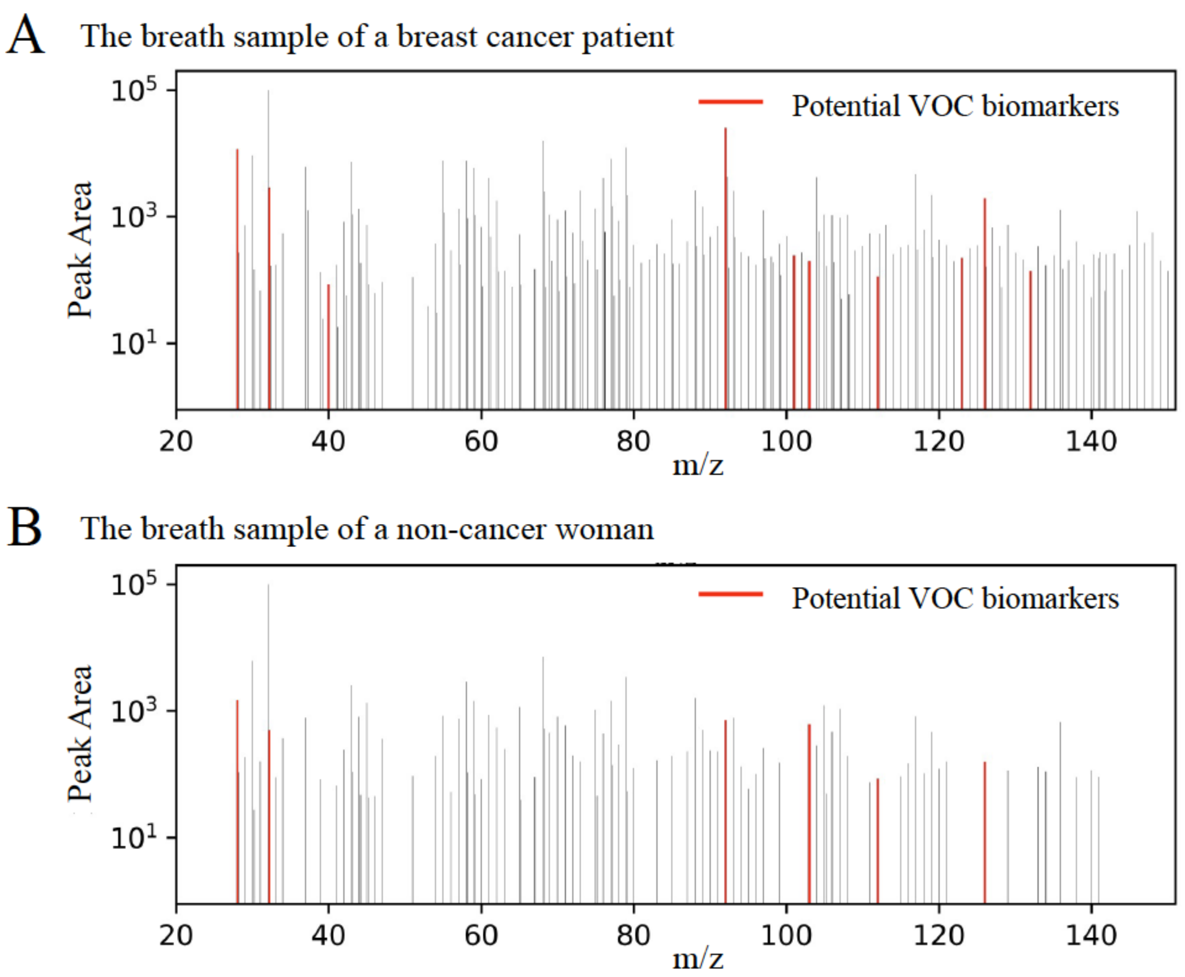

Figure S2. Examples of Mass Spectrums in the Breath Samples from a Breast Cancer Patient and a Non-cancer Woman.

The peak area was then computed for each VOC ion in breath samples. Spectrum peaks of the breast cancer patients and non-cancer women showed distinct patterns between the m/z ranging from 20 to 140. Ten optimal VOC features with the most significant differences between the cases and controls were high-lighted in red lines.

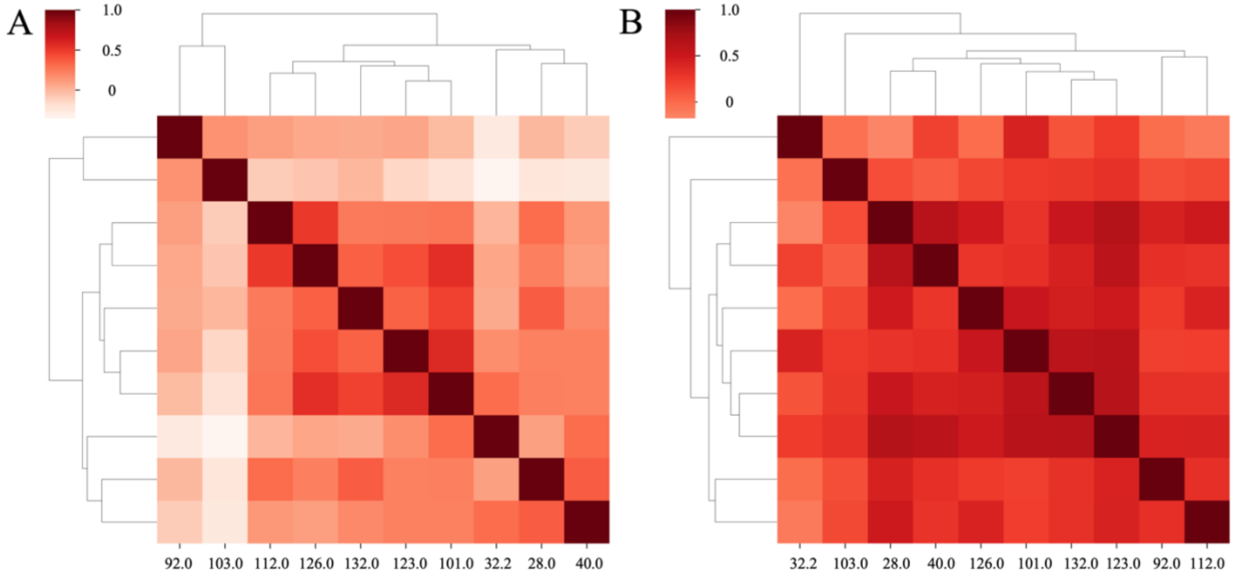

Figure S3. The correlation analysis of VOC ions among the control group (A) and the breast cancer group (B).

The different composition of VOCs between patients with breast cancer and non-cancer controls was demonstrated by correlation analyses, which suggested that breast cancer patients have distinct VOCs correlation modules.

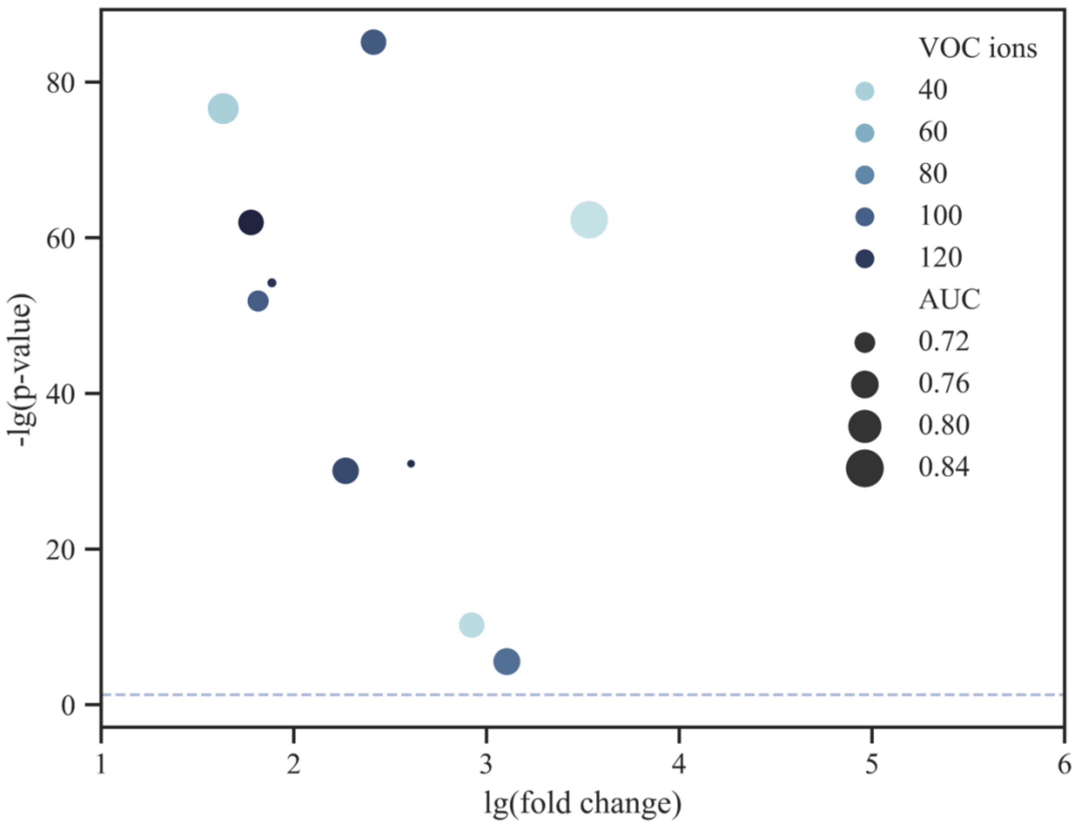

Figure S4. The volcano plot shows the group difference and in breath VOC ions peak area between breast cancer patients and controls.

Significant fold changes and diagnostic performances between the patients with breast cancer and non-cancer controls were identified in the 10 VOC ions.

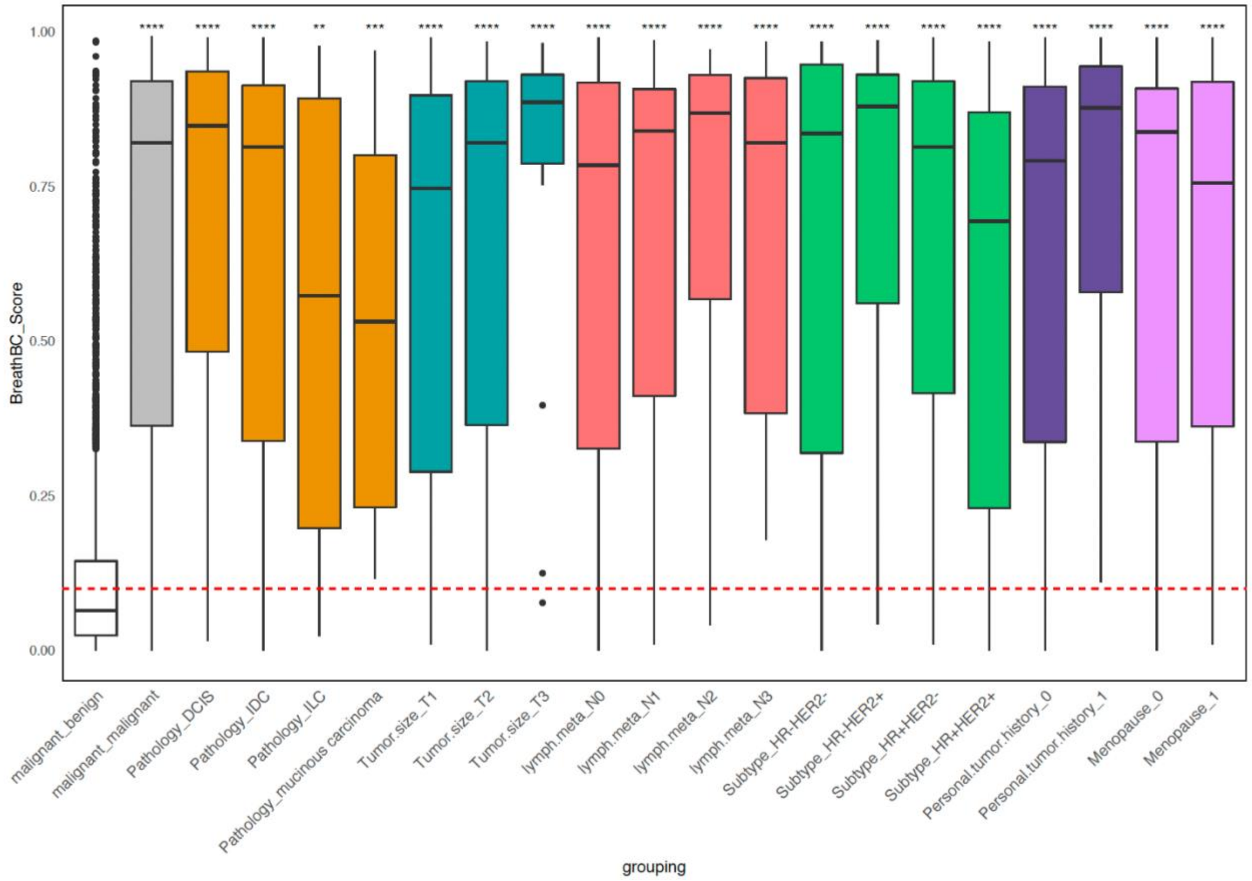

Figure S5. Evaluating Clinical and Pathologic Characteristics with the BreathBC Score. Overall, the BreathBC scores were higher in patients with breast cancer than among the controls, regardless of pathology, tumor size, lymph node status, molecular subtype, personal cancer history, and menopause status (all $p<0.01$). * $p \le 0.05$; ** $p \le 0.01$; *** $p \le 0.001$; **** $p \le 0.0001$. Abbreviation: DCIS, ductal carcinoma *in situ*; IDC, invasive ductal carcinoma; ILC, invasive lobular carcinoma; T, tumor; N, lymph node.

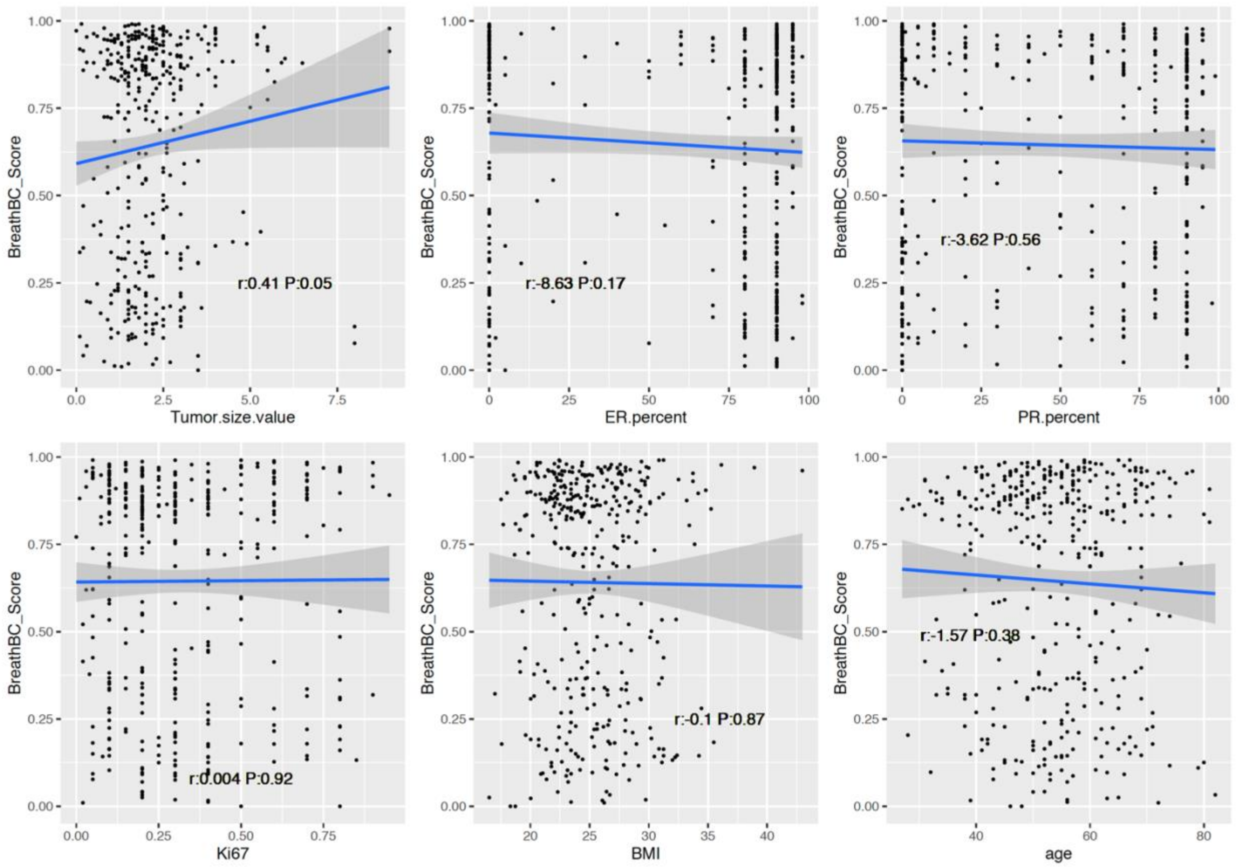

Figure S6. The BreathBC Score Correlates Tumor Size, Estrogen Receptor (ER), Progesterone Receptor (PR), Ki-67, Body Mass Index (BMI), and Age.

The BreathBC scores were collinear with tumor size (r=0.41, p=0.05) and negatively correlated to estrogen and progesterone receptor status (r=−8.63 and −3.62, p=0.17 and 0.56, respectively).

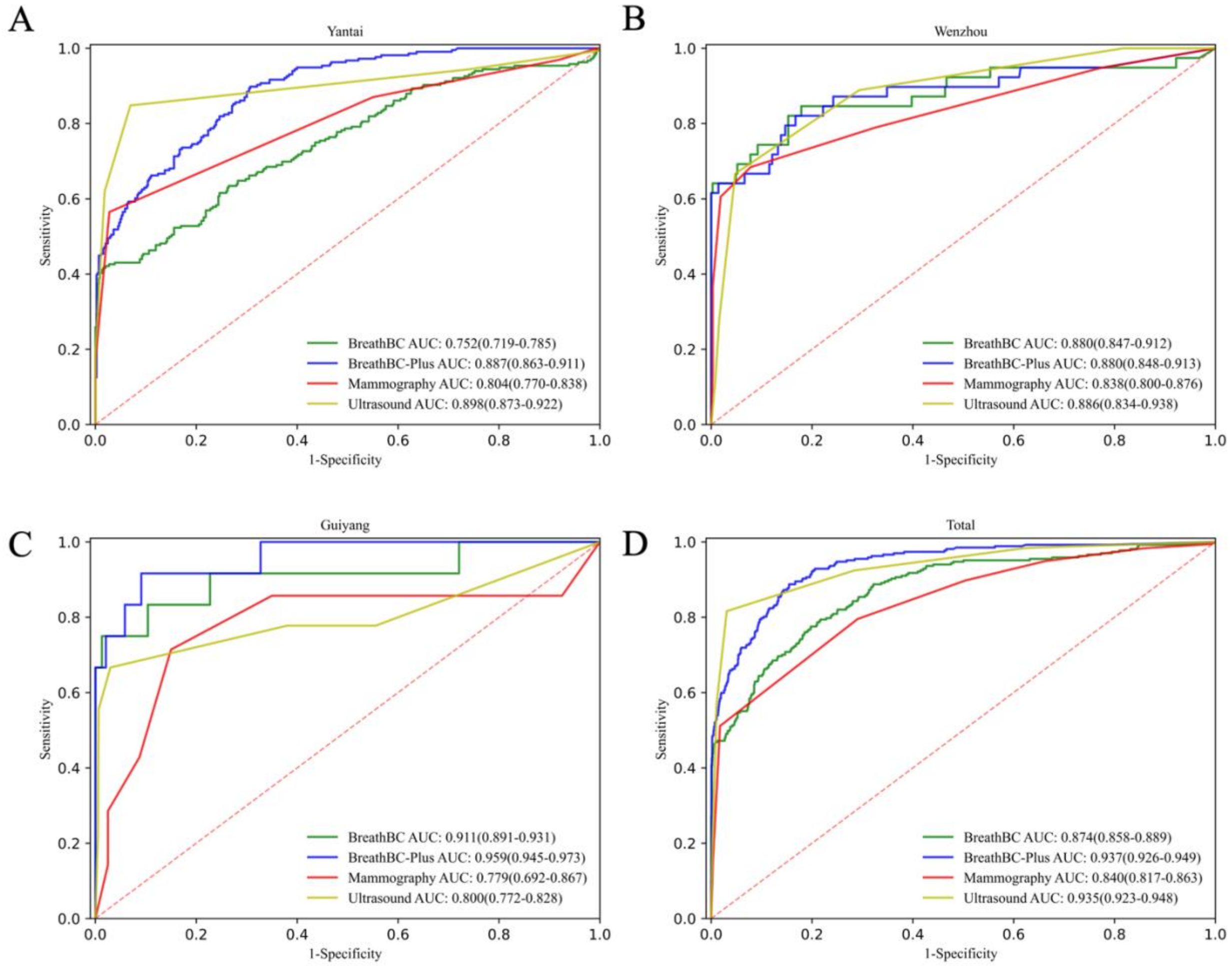

Figure S7. Receiver Operating Characteristic (ROC) Curves and the Associated Areas under Curves (AUCs) of the Diagnostic Prediction Model Using the Breath-based Screening Methods (BreathBC and BreathBC plus) and the Imaging-based Screening Methods (Mammography and Ultrasound) in Yantai, Wenzhou and Guiyang cohort.

When compared with traditional imaging-based screening methods, the diagnostic performance of the BreathBC was superior to that of mammography in the external validation cohorts (Table S5). However, the performance of the BreathBC model was still not comparable to that of ultrasound (p=1.28×10−13). Furthermore, the diagnostic performance of BreathBC-Plus was superior to that of mammography and ultrasound in the external validation cohorts (AUC [95%CI]: 0.94 [0.93–0.95] for BreathBC-Plus vs. 0.84 [0.82–0.86] for mammography and 0.94 [0.92–0.95] for ultrasound, $p=2.88\times10^{-46}$ and 1.46×10^{-32}, respectively; Table S5).

Table S1. Demographic Characteristics of Participants at Baseline.

Discovery Set		Breast cancer（n=216）	Controls(n=2959)	*P* value
Age	mean ± SD	54.33±11.06	49.71±8.53	6.82×10^{-9}
BMI	mean ± SD	24.56±3.55	24.86±3.25	0.08
Family cancer history	With	31.16% (67/215)	14.80% (405/2737)	2.89×10^{-10}
	Without	68.84% (148/215)	85.20% (2332/2737)	
Family BC history	With	10.23% (22/215)	3.14% (86/2737)	9.70×10^{-8}
	Without	89.77% (193/215)	96.86% (2651/2737)	
Personal cancer history	With	15.81% (34/215)	0.69% (19/2740)	3.19×10^{-58}
	Without	84.19% (181/215)	99.31% (2721/2740)	
Menopause	Premenopausal	38.89% (84/216)	84.41% (758/898)	2.00×10^{-44}
	Postmenopausal	61.11% (132/216)	15.59% (140/898)	
External Validation Set		Breast cancer（n=249）	Controls(n=1545)	*P* value
Age	mean ± SD	53.61±11.12	43.14±9.69	2.63×10^{-34}
BMI	mean ± SD	25.57±3.97	22.95±3.32	1.63×10^{-19}
Family cancer history	With	24.10% (60/249)	12.94% (200/1545)	3.00×10^{-6}
	Without	75.10% (187/249)	87.06% (1345/1545)	
Family BC history	With	4.02% (10/249)	3.69% (57/1545)	0.78
	Without	95.18% (237/249)	96.31% (1488/1545)	
Personal cancer history	With	6.02% (15/249)	2.33% (36/1545)	1.02×10^{-3}
	Without	93.17% (232/249)	97.67% (1509/1545)	
Menopause	Premenopausal	45.19% (108/239)	59.08% (732/1239)	7.20×10^{-5}
	Postmenopausal	54.81% (131/239)	40.92% (507/1239)	

Abbreviation: SD, standard deviation; BMI, body mass index; BC, breast cancer.

Table S2. Demographic Characteristics of Participants in the External Validation Cohorts.

Yantai Cohort		Breast cancer（n=199）	Controls(n=441)	*P* value
Age	mean ± SD	54.54±11.43	40.94±10.99	$1.63×10^{-40}$
BMI	mean ± SD	26.19±3.90	23.54±3.31	$3.64×10^{-15}$
Family cancer history	With	29.65% (59/199)	16.55% (73/441)	$1.51×10^{-4}$
	Without	70.35% (140/199)	83.45% (368/441)	
Family BC history	With	4.52% (9/199)	4.08% (18/441)	0.80
	Without	95.48% (190/199)	95.92% (423/441)	
Personal cancer history	With	7.54% (15/199)	4.31% (19/441)	0.09
	Without	92.46% (184/199)	95.69% (422/441)	
Menopause	Premenopausal	41.41% (82/198)	16.78% (74/441)	$2.03×10^{-11}$
	Postmenopausal	58.59% (116/198)	83.22% (367/441)	
Wenzhou Cohort		Breast cancer（n=38）	Controls(n=322)	*P* value
Age	mean ± SD	48.58±8.58	47.03±7.80	0.27
BMI	mean ± SD	22.95±3.25	22.44±2.93	0.34
Family cancer history	With	2.63% (1/38)	8.70% (28/322)	0.34*
	Without	97.37% (37/38)	91.30% (294/322)	
Family BC history	With	2.63% (1/38)	3.11% (10/322)	1.00*
	Without	97.37% (37/38)	96.89% (312/322)	
Personal cancer history	With	0% (0/38)	4.97% (16/322)	0.39*
	Without	100% (38/38)	95.03% (306/322)	
Menopause	Premenopausal	67.65% (23/34)	80.76% (235/291)	0.07
	Postmenopausal	32.35% (11/34)	19.24% (56/291)	
Guiyang Cohort		Breast cancer（n=12）	Controls(n=782)	*P* value
Age	mean ± SD	53.11±7.94	42.85±9.12	$8.17×10^{-4}$
BMI	mean ± SD	22.89±2.78	22.83±3.44	0.96
Family cancer history	With	0% (0/12)	12.66% (99/782)	0.38*
	Without	100% (12/12)	87.34% (683/782)	
Family BC history	With	0% (0/12)	3.71% (29/782)	1.00*
	Without	100% (12/12)	96.29% (753/782)	
Personal cancer history	With	0% (0/12)	0.13% (1/782)	1.00*
	Without	100% (12/12)	99.87% (781/782)	
Menopause	Premenopausal	42.86% (3/7)	83.43% (423/507)	0.02*
	Postmenopausal	57.14% (4/7)	16.57% (84/507)	

*Fisher's Exact Test

Abbreviation: SD, standard deviation; BMI, body mass index; BC, breast cancer.

Table S3. Clinical Characteristics of Breast Cancer Patients in this Multi-Center Study.

Characteristics	Discovery Set	External Validation Set		
	Beijing Cohort (N=216)	Yantai Cohort (N=199)	Wenzhou Cohort (N=38)	Guiyang Cohort (N=12)
Age, yr/o [a]	54.33±11.06	54.54±11.43	48.58±8.58	53.11±7.94
Malignancy rate	6.82% (216/3167)	31.09% (199/640)	10.56% (38/360)	1.51% (12/794)
Histology [b]				
IDC	83.33% (180/216)	78.89% (157/199)	63.16% (24/38)	75.00% (9/12)
DCIS	9.72% (21/216)	10.55% (21/199)	28.95% (11/38)	8.33% (1/12)
Lobular	2.31% (5/216)	3.52% (7/199)	5.26% (2/38)	0% (0/12)
Mucinous	2.31% (5/216)	4.02% (8/199)	2.63% (1/38)	16.67% (2/12)
Others [c]	1.39% (3/216)	3.02% (6/199)	0% (0/38)	0% (0/12)
Unknown	0.93% (2/216)	0% (0/199)	0% (0/38)	0% (0/12)
Tumor size[b]				
≤2 cm	50.93% (110/216)	54.27% (108/199)	71.05% (28/38)	25.00% (3/12)
2-5 cm	36.11% (78/216)	42.71% (85/199)	18.42% (7/38)	50.00% (6/12)
≥5 cm	5.56% (12/216)	2.01% (4/199)	2.63% (1/38)	25.00% (3/12)
Unknown	7.41% (16/216)	1.00% (2/199)	5.26% (2/38)	0% (0/12)
Lymph nodes status [b]				
Positive	38.89% (84/216)	26.63% (53/199)	31.58% (12/38)	41.67% (5/12)
Negative	58.80% (127/216)	73.36% (144/199)	68.42% (26/38)	58.33% (7/12)
Unknown	2.31% (5/216)	1.00% (2/199)	0% (0/38)	0% (0/12)
Stage[b, d]				
0/DCIS	9.72% (21/216)	10.55% (21/199)	28.95% (11/38)	8.33% (1/12)
I	31.48% (68/216)	35.68% (71/199)	39.47% (15/38)	8.33% (1/12)
II	36.11% (78/216)	48.24% (96/199)	21.05% (8/38)	50.00% (6/12)
III	19.91% (43/216)	5.03% (10/199)	10.53% (4/38)	25.00% (3/12)
IV	0.93% (2/216)	0.50% (1/199)	0% (0/38)	8.33% (1/12)
Unknown	1.85% (4/216)	0% (0/199)	0% (0/38)	0% (0/12)
Molecular subtype[b]				
HR+/HER2-	61.57% (133/216)	65.33% (130/199)	47.37% (18/38)	83.33% (10/12)
HR-/HER2+	12.04% (26/216)	10.55% (21/199)	10.53% (4/38)	16.67% (2/12)
HR+/HER2+	11.57% (25/216)	11.56% (23/199)	15.79% (6/38)	0% (0/12)
TNBC	9.72% (21/216)	7.04% (14/199)	5.26% (2/38)	0% (0/12)
Unknown	5.09% (11/216)	5.53% (11/199)	21.05% (8/38)	0% (0/12)
Ki67 [b]				
≤30%	67.13% (145/216)	62.31% (124/199)	63.16% (24/38)	75.00% (9/12)
> 30%	30.09% (65/216)	35.18% (70/199)	26.32% (10/38)	25.00% (3/12)
Unknown	2.78% (6/216)	2.51% (5/199)	10.53% (4/38)	0% (0/12)

[a] Data shown as the mean ± standard deviation.

[b] Data shown as No. (%).

[c] Others include metaplastic cancer, sieve cancer, Paget's disease, micropapillary cancer, secretory cancer, and tubule cancer.

[d] Clinical staging was determined according to the eighth edition of the classification for breast cancer of the American Joint Commission of Cancer.[20]

Abbreviation: IDC, invasive ductal carcinoma; DCIS, ductal carcinoma *in situ*; HR, hormone receptor; HER2, human epidermal growth factor receptor 2; TNBC, triple-negative breast cancer.

Table S4. Comparisons of Spectrum Peak Area of VOCs in Patients with Breast Cancer and Non-Cancer Controls in All the Participants.

m/z	Tendency in breast cancer	Potential VOCs	Breast cancer (Mean ± SD)	Control (Mean ± SD)	*p*-value
28	↑	ethylene	3725.1±2328.8	1841.4±208.5	1.39×10^{-40}
40	↑	propyne or fragment ion	38.4±20.3	3.3±8.4	7.59×10^{-109}
92	↓	isoprene	492.6±377.0	1029.7±434.6	3.60×10^{-88}
132	↑	l-asparagine	43.8±42.8	0.0±0.0	5.02×10^{-57}
123	↑	nicotinic acid	64.3±53.6	4.0±13.0	6.78×10^{-65}
112	↑	uracil	232.3±147.0	122.9±71.5	1.78×10^{-35}
103	↓	protonated ion of pivalic acid	120.1±65.5	299.5±150.0	2.45×10^{-200}
126	↑	thymine	500.9±278.5	252.7±136.9	1.23×10^{-46}
101	↑	protonated ion of hexanone	76.3±43.0	17.3±28.9	9.36×10^{-84}
32.2	↑	methanol	1589.5±1025.4	805.7±879.5	2.20×10^{-36}

Abbreviation: VOC, volatile organic compound; SD, standard deviation.

Table S5. The Prediction Accuracy of the BreathBC and BreathBC-Plus in Multi-Center Validation Cohort.

	Internal validation cohort	Test cohort	External validation cohorts
BreathBC			
AUC (95%CI)	0.96 (0.942-0.97)	0.95 (0.93-0.96)	0.87 (0.86-0.89)
Sensitivity	97.67% (42/43)	93.85% (61/65)	92.37% (230/249)
Specificity	61.49% (364/592)	61.75% (549/889)	60.45% (934/1545)
BreathBC-plus			
AUC (95%CI)	0.98 (0.97-0.99)	0.97 (0.96-0.98)	0.94 (0.93-0.95)
Sensitivity	97.67% (42/43)	96.92% (63/65)	89.16% (222/249)
Specificity	77.36% (458/592)	76.60% (681/889)	87.70% (1355/1545)

* The cut-off values for the BreathBC and BreathBC-Plus models are both 0.10.

Abbreviation: AUC, area under the curve; CI, confidence interval.

Table S6. Summary of Volatile Organic Compounds (VOCs) Studies on the Breast Cancer

First Author, Year, Ref	Country /Region	Method	VOC Biomarkers or Selected Method	Study Design	Validation	Participants Number (Cases vs. Controls)	Sensitivity/Specificity	AUC
Philips, 2003[18]	US	GC-MS	markers of oxidative stress	case-control	cross-validation	51 BC vs. 42 HC	88.2%/73.8%	0.91
						51 BC vs. 50 Benign	60.8%/82.0%	NA
Philips, 2006[19]	US	GC-MS	5 VOC markers	case-control	random assignment	51 vs. 42	93.80%/84.60%	0.90
Philips, 2010[20]	Australia	GC-MS	the VOCs in the top ten time slices ranked according their C-statistic values	case-control	randomly allocated to training sets or test sets	54 vs. 204	87.50%/79.70%	0.87
Philips, 2014[21]	US; Netherlands	GC-MS	C-statistic values and Monte Carlo simulations	case-control pilot		35 vs. 79	75.8%/74.0%	0.67
Phillips, 2017[22]	Australia	GS-MS	multiple Monte Carlo simulations	case-control		54 vs. 204	NA/NA	0.77
Phillips, 2018[23]	US; Mexico	GC-MS	multiple Monte Carlo simulations	case-control		54 vs. 124	NA/NA	0.77
Sinues, 2015[24]	Italy	SESI–MS	Wilcoxon–Mann–Whitney test, bootstrap feature selection	case-control		14 vs. 11	93%/91%	NA
Li, 2014[25]	China	GC-MS	Kruskal-Wallis one-way ANOVA test	case-control		22 vs. 24	68.20%/91.70%	0.93
Wang, 2014[26]	China	GC-MS	PLS-DA, PCA	case-control		85 vs. 45	NA	NA
Zhang, 2020[27]	China	GC-MS	overlap between significantly different VOCs in BC vs HC	prospective cohort		71 vs. 78	93.59%/71.62%	0.92
Yang, 2021[28]	Taiwan, China	Electronic noses		case-control	randomly divided 20% into a test set	351 vs. 88	86%/97%	0.99

Abbreviation: VOCs, volatile organic compounds; AUC, area under the curve; US: United States of America; GC-MS: gas chromatography-mass spectrometry; BC, breast cancer; HC: healthy control; NA: not applied; SESI–MS: secondary electrospray

ionization-mass spectrometry; ANOVA: analysis of variance; PLS-DA: partial leastsquares discriminant analysis; PCA: principal component analysis.

Table S7. The Prediction Accuracy of the BreathBC and BreathBC-Plus in External Validation Cohorts.

	Yantai cohort	Wenzhou cohort	Guiyang cohort	Total
BreathBC				
AUC (95%CI)	0.75 (0.72-0.79)	0.88 (0.85-0.91)	0.91 (0.89-0.93)	0.87 (0.86-0.89)
Sensitivity	92.46% (184/199)	94.74% (36/38)	83.33% (10/12)	92.37% (230/249)
Specificity	28.34% (125/441)	50.93% (164/322)	82.48% (645/782)	60.45% (934/1545)
BreathBC-plus				
AUC (95%CI)	0.88 (0.87-0.90)	0.88 (0.85-0.91)	0.96 (0.95-0.97)	0.94 (0.93-0.95)
Sensitivity	90.95% (181/199)	81.58% (31/38)	83.33% (10/12)	89.16% (222/249)
Specificity	66.21% (292/441)	90.99% (293/322)	98.47% (770/782)	87.70% (1355/1545)

* The cut-off values for the BreathBC and BreathBC-Plus models are both 0.10.

Abbreviation: AUC, area under the curve; CI, confidence interval.

Table S8. The Detection Rates of the BreathBC and BreathBC-Plus in the External Validation Cohorts.

	Yantai cohort	Wenzhou cohort	Guiyang cohort	Total
BreathBC				
Stage[a, b]				
0/DCIS	100% (21/21)	90.91% (10/11)	100% (1/1)	96.97% (32/33)
I	92.86% (66/71)	93.33% (14/15)	100% (1/1)	93.10% (81/87)
II	89.58% (86/96)	100% (8/8)	83.33% (5/6)	90.00% (99/110)
III	100% (10/10)	100% (4/4)	66.67% (2/3)	94.12% (16/17)
IV	100% (1/1)	NA (0)	100% (1/1)	100% (2/2)
Total	92.46% (184/199)	94.74% (36/38)	83.33% (10/12)	92.37% (230/249)
BreathBC-plus				
Stage[a, b]				
0/DCIS	100% (21/21)	81.82% (10/11)	100% (1/1)	96.97% (32/33)
I	88.73% (63/71)	73.33% (11/15)	0% (0/1)	85.06% (74/87)
II	89.58%% (86/96)	87.50% (7/8)	100% (6/6)	90.00% (99/110)
III	100% (10/10)	75.00% (3/4)	66.67% (2/3)	88.24% (15/17)
IV	100% (1/1)	NA (0)	100% (1/1)	100% (2/2)
Total	90.95% (181/199)	81.58% (31/38)	83.33% (10/12)	89.16% (222/249)

[a] Data shown as No. (%).

[b] Clinical staging was determined according to the eighth edition of the classification for breast cancer of the American Joint Commission of Cancer.[20]

* The cut-off values for the BreathBC and BreathBC-Plus models are both 0.10.

Abbreviation: NA, not applicable.

Table S9. Diagnostic Performance of the Breath-based Screening Methods (BreathBC and BreathBC-plus) and the Imaging-based Screening Methods (Mammography and Ultrasound) in the Multi-Center Validation Cohorts.

External validation	AUC (95%CI) *Breast cancer: Healthy control*				*P* value*			
	1.BreathBC	2.BreathBC Plus	3.Mammography	4.Ultrasonography	1 vs.3	1 vs.4	2 vs.3	2 vs.4
Yantai cohort	0.75 (0.72-0.79) *199:441*	0.89 (0.86-0.91) *199:441*	0.80 (0.77-0.84) *131:396*	0.90 (0.87-0.92) *158:432*	**3.59×10^{-17}**	**1.00×10^{-13}**	**3.79×10^{-55}**	**8.40×10^{-52}**
Wenzhou cohort	0.88 (0.85-0.91) *38:322*	0.88 (0.85-0.91) *38:322*	0.84 (0.80-0.88) *38:319*	0.89 (0.83-0.94) *18:126*	**2.84×10^{-8}**	**1.95×10^{-4}**	**2.30×10^{-9}**	**1.01×10^{-4}**
Guiyang cohort	0.91 (0.89-0.93) *12:782*	0.96 (0.95-0.97) *12:782*	0.78 (0.69-0.87) *7:80*	0.80 (0.78-0.83) *9:770*	0.221	**2.10×10^{-2}**	0.134	**9.50×10^{-4}**
Total	0.87(0.86-0.89) *249:1545*	0.94(0.93-0.95) *249:1545*	0.84(0.82-0.86) *176:795*	0.94(0.92-0.95) *185:1328*	**4.06×10^{-24}**	**1.28×10^{-13}**	**2.88×10^{-46}**	**1.46×10^{-32}**

**P* value was calculated using the Delong's method.

Abbreviation: AUC, area under the curve; CI, confidence interval.

References

1. Zhang X, Yang L, Liu S, et al. Evaluation of Different Breast Cancer Screening Strategies for High-Risk Women in Beijing, China: A Real-World Population-Based Study. *Frontiers in Oncology*. 2021;11. Accessed August 27, 2022. https://www.frontiersin.org/articles/10.3389/fonc.2021.776848

2. Wang Y, Chen H, Li N, et al. Ultrasound for Breast Cancer Screening in High-Risk Women: Results From a Population-Based Cancer Screening Program in China. *Frontiers in Oncology*. 2019;9. Accessed August 27, 2022. https://www.frontiersin.org/articles/10.3389/fonc.2019.00286

3. Pm B, Jb R, De B, et al. STARD 2015: an updated list of essential items for reporting diagnostic accuracy studies. *BMJ (Clinical research ed)*. 2015;351. doi:10.1136/bmj.h5527

4. American College of Radiology. *ACR BI-RADS Atlas: Breast Imaging Reporting and Data System*. 5th ed. Reston, Virginia; 2013.

5. Spak DA, Plaxco JS, Santiago L, Dryden MJ, Dogan BE. BI-RADS® fifth edition: A summary of changes. *Diagnostic and Interventional Imaging*. 2017;98(3):179-190. doi:10.1016/j.diii.2017.01.001

6. Waks AG, Winer EP. Breast Cancer Treatment: A Review. *JAMA*. 2019;321(3):288-300. doi:10.1001/jama.2018.19323

7. American Joint Committee on Cancer (AJCC). *AJCC Cancer Staging Manual*. 8th ed. New York: Springer; 2017.

8. Wang Y, Jiang J, Hua L, et al. High-Pressure Photon Ionization Source for TOFMS and Its Application for Online Breath Analysis. *Anal Chem*. 2016;88(18):9047-9055. doi:10.1021/acs.analchem.6b01707

9. Meng S, Li Q, Zhou Z, et al. Assessment of an Exhaled Breath Test Using High-Pressure Photon Ionization Time-of-Flight Mass Spectrometry to Detect Lung Cancer. *JAMA Netw Open*. 2021;4(3):e213486. doi:10.1001/jamanetworkopen.2021.3486

10. Lee G, Gommers R, Waselewski F, Wohlfahrt K, O'Leary A. PyWavelets: A Python package for wavelet analysis. *JOSS*. 2019;4(36):1237. doi:10.21105/joss.01237

11. Kuo TC, Tan CE, Wang SY, et al. Human Breathomics Database. *Database (Oxford)*. 2020;2020:baz139. doi:10.1093/database/baz139

12. Breiman L. Random Forests. *Machine Learning*. 2001;45(1):5-32. doi:10.1023/A:1010933404324

13. DeLong ER, DeLong DM, Clarke-Pearson DL. Comparing the areas under two or more correlated receiver operating characteristic curves: a nonparametric approach. *Biometrics*. 1988;44(3):837-845.

14. Sun X, Xu W. Fast Implementation of DeLong's Algorithm for Comparing the Areas Under Correlated Receiver Operating Characteristic Curves. *IEEE Signal Processing Letters*. 2014;21(11):1389-1393. doi:10.1109/LSP.2014.2337313

15. Liu J, Zhao H, Huang Y, et al. Genome-wide cell-free DNA methylation analyses improve accuracy of non-invasive diagnostic imaging for early-stage breast cancer. *Molecular Cancer*. 2021;20(1):36. doi:10.1186/s12943-021-01330-w

16. Hanley JA, McNeil BJ. A method of comparing the areas under receiver operating characteristic curves derived from the same cases. *Radiology*. 1983;148(3):839-843. doi:10.1148/radiology.148.3.6878708

17. Obuchowski NA, McClish DK. Sample size determination for diagnostic accuracy studies involving binormal ROC curve indices. *Stat Med*. 1997;16(13):1529-1542. doi:10.1002/(sici)1097-0258(19970715)16:13<1529::aid-sim565>3.0.co;2-h

18. Phillips M, Cataneo RN, Ditkoff BA, et al. Volatile Markers of Breast Cancer in the Breath. *Breast Journal*. 2003;9(3):184-191. doi:10.1046/j.1524-4741.2003.09309.x

19. Phillips M, Cataneo RN, Ditkoff BA, et al. Prediction of breast cancer using volatile biomarkers in the breath. *Breast Cancer Res Treat*. 2006;99(1):19-21. doi:10.1007/s10549-006-9176-1

20. Phillips M, Cataneo RN, Saunders C, Hope P, Schmitt P, Wai J. Volatile biomarkers in the breath of women with breast cancer. *J Breath Res*. 2010;4(2):026003. doi:10.1088/1752-7155/4/2/026003

21. Phillips M, Beatty JD, Cataneo RN, et al. Rapid Point-Of-Care Breath Test for Biomarkers of Breast Cancer and Abnormal Mammograms. Aziz SA, ed. *PLoS ONE*. 2014;9(3):e90226. doi:10.1371/journal.pone.0090226

22. Phillips M, Cataneo RN, Lebauer C, Mundada M, Saunders C. Breath mass ion biomarkers of breast cancer. *J Breath Res*. 2017;11(1):016004. doi:10.1088/1752-7163/aa549b

23. Phillips M, Cataneo RN, Cruz-Ramos JA, et al. Prediction of breast cancer risk with volatile biomarkers in breath. *Breast Cancer Res Treat*. 2018;170(2):343-350. doi:10.1007/s10549-018-4764-4

24. Martinez-Lozano Sinues P, Landoni E, Miceli R, et al. Secondary electrospray ionization-mass spectrometry and a novel statistical bioinformatic approach identifies a cancer-related profile in exhaled breath of breast cancer patients: a pilot study. *J Breath Res*. 2015;9(3):031001. doi:10.1088/1752-7155/9/3/031001

25. Li J, Peng Y, Liu Y, et al. Investigation of potential breath biomarkers for the early diagnosis of breast cancer using gas chromatography–mass spectrometry. *Clinica Chimica Acta*. 2014;436:59-67. doi:10.1016/j.cca.2014.04.030

26. Wang C, Sun B, Guo L, et al. Volatile Organic Metabolites Identify Patients with Breast Cancer, Cyclomastopathy and Mammary Gland Fibroma. *Sci Rep*. 2015;4(1):5383. doi:10.1038/srep05383

27. Zhang Y, Guo L, Qiu Z, Lv Y, Chen G, Li E. Early diagnosis of breast cancer from exhaled breath by gas chromatography-mass spectrometry (GC/MS) analysis: A prospective cohort study. *J Clin Lab Anal*. 2020;34(12). doi:10.1002/jcla.23526

28. Yang HY, Wang YC, Peng HY, Huang CH. Breath biopsy of breast cancer using sensor array signals and machine learning analysis. *Sci Rep*. 2021;11(1):103. doi:10.1038/s41598-020-80570-0
